# Teaching Methodologies for First Aid in Physical Education in Secondary Schools: A Systematic Review

**DOI:** 10.3390/healthcare13101112

**Published:** 2025-05-10

**Authors:** José María Parada-Espinosa, Sonia Ortega-Gómez, Manuel Ruiz-Muñoz, Jara González-Silva

**Affiliations:** 1 Faculty of Education Sciences, University of Cadiz, 11519 Puerto Real, Spain; josemaria.paradaespinosa@alum.uca.es; 2 MOVE-IT Research Group, Department of Physical Education, Faculty of Education Sciences, Instituto de Investigación e Innovación Biomédica de Cádiz (INiBICA), Universidad de Cádiz, 11009 Cádiz, Spain; manuel.ruizmunoz@alum.uca.es; 3 Faculty of Education, University of Sevilla, 41013 Sevilla, Spain

**Keywords:** physical education, first aid, active methodologies, gamification, simulation, knowledge retention

## Abstract

**Background**: First aid training in secondary education enhances emergency preparedness and supports public health. Despite its inclusion in many school curricula, there is no consensus on the most effective teaching methodologies. This systematic review aims to compare instructional strategies used in first aid training during Physical Education and evaluate their impact on students’ knowledge, practical skills, and confidence. **Methods**: A systematic review was conducted in accordance with PRISMA 2020 guidelines. Six databases (SCOPUS, Web of Science, ERIC, DIALNET, MEDLINE, and PsycINFO) were searched up to December 2024. Eligible studies were quasi-experimental or observational, involved students aged 11–18, and focused on first aid instruction within Physical Education. Methodological quality was assessed using the PEDro scale. **Results**: Eleven studies with a total of 3069 students aged 11–18 were included. Active and technology-based methodologies outperformed traditional approaches, improving knowledge acquisition (10.2–30.5%) and practical skill development (18.6–42.3%). Long-term retention ranged from 14.2% to 45.8%, with longer interventions yielding better outcomes. Gamification, simulations, and peer learning improved CPR quality and boosted student confidence. However, most studies assessed only short-term outcomes, limiting conclusions about sustained learning. **Conclusions**: Active methodologies, particularly gamification, simulation, and cooperative learning, enhance knowledge retention, practical skills, and confidence in providing first aid. Although the results were consistently positive, methodological heterogeneity and limited long-term follow-up reduce their generalizability. Further high-quality, longitudinal research is needed to identify the most effective and sustainable strategies. These findings support integrating first aid training into Physical Education as a public health initiative to strengthen emergency preparedness in schools.

## 1. Introduction

First aid consists of the immediate assistance provided to individuals in emergency situations such as choking, nosebleeds, fainting, sprains, fractures, cuts, or falls. When administered promptly and effectively, it can significantly reduce mortality and mitigate potential complications [1,2,3].

Teaching first aid from an early age is considered a strategic approach to enhancing emergency response capacity and fostering a culture of prevention and safety [4,5,6]. Schools are particularly well-suited for this purpose, providing a structured and widespread platform for instruction with clear public health benefits [3,7,8,9].

Global initiatives, such as Kids Save Lives, endorsed by the European Resuscitation Council (ERC) [10] and the World Health Organization (WHO), advocate for the mandatory inclusion of cardiopulmonary resuscitation (CPR) training in school curricula. These programs have been shown to increase bystander CPR rates and improve survival outcomes [11,12]. For example, Denmark experienced a twofold increase in bystander CPR interventions and a threefold increase in survival following the nationwide implementation of CPR training in schools [13,14].

Countries such as Germany and the United Kingdom have also integrated CPR and first aid education into their school systems through legislative initiatives [3]. In Spain, although these competencies are formally included in the curriculum, particularly within Physical Education (PE), their implementation remains limited due to insufficient teacher training, a lack of adapted educational resources, and the absence of context-appropriate teaching methodologies [3,4,11].

A wide range of instructional strategies has been employed to teach CPR and first aid in school settings, including traditional instructor-led lessons and more participatory approaches (e.g., simulation-based learning, peer instruction, digital platforms, gamification, and augmented reality) [3,4,5].

Current evidence supports the use of active and participatory methods, which are associated with improved skill acquisition, knowledge retention, and student confidence [3,11,15,16,17]. In contrast, traditional approaches typically rely on lecture-based instruction with limited hands-on engagement [18,19].

However, despite these promising results, there is no consensus regarding the optimal implementation of these methods in secondary school contexts. The variability in study designs, outcome measures, and intervention durations complicates cross-study comparisons and the formulation of best practices [11,20]. Therefore, there is a pressing need for rigorously designed comparative studies specifically targeting secondary school PE settings to determine the most effective strategies for long-term retention and real-life applicability in emergency scenarios [3,9,17,21].

Several systematic reviews have examined CPR and first aid instruction in schools [1,11], but most focus on general populations or mixed educational stages, without specifically addressing Physical Education (PE) in secondary schools. For instance, studies by Abelairas-Gómez [3,22], Beck [23], Beskind [24], and Plant and Taylor [25] explored training frequency, peer education, video-based CPR, and general strategies, respectively. These reviews often include heterogeneous samples, lack methodological comparisons, and offer limited recommendations for PE contexts. In contrast, our review targets secondary school PE, providing a structured analysis of teaching strategies, duration, assessment tools, and outcomes, with practical guidance for educators and policymakers.

Focusing on secondary students is strategic, as they have greater maturity to learn and retain life-saving skills before leaving compulsory education. Supporting evidence includes Watanabe et al. [26], who found long-term gains from a 45 min CPR session, and Miró et al., who confirmed the success of a hybrid PE–healthcare model in Spain.

This systematic review addresses that gap by comparing traditional and innovative teaching methodologies for first aid instruction within the PE context, with the objective of identifying the most effective pedagogical strategies for enhancing students’ emergency response competencies and supporting the curricular inclusion of first aid training.

## 2. Materials and Methods

**Figure 1:**
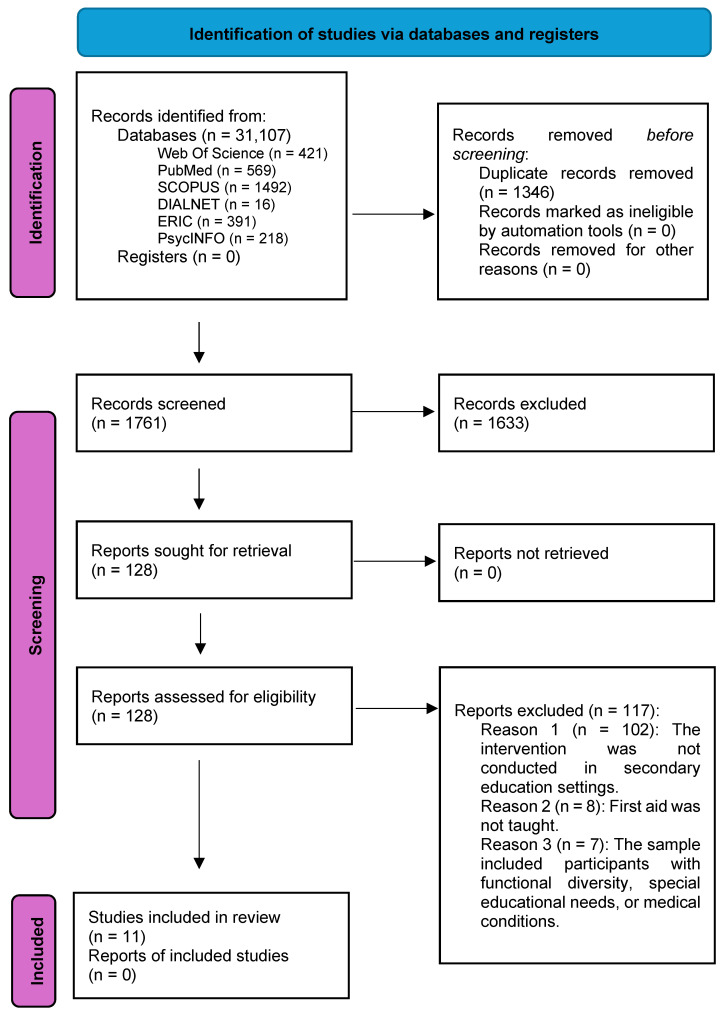
Flowchart of the article selection process in the systematic review according to PRISMA guidelines, Modified from Moher et al. [29]. Preferred reporting items for systematic reviews and meta-analyses: the PRISMA statement.

This systematic review was conducted in accordance with the PRISMA 2020 [27,28] guidelines, ensuring methodological rigor in the identification, selection, analysis, and synthesis of studies. A completed PRISMA 2020 checklist is available in Supplementary Table S1, and the PRISMA flow diagram detailing the study selection process is included in Figure 1. Additionally, the PRISMA 2020 for Abstracts Checklist is provided in Supplementary Table S2. The protocol of this systematic review was registered in the PROSPERO International Prospective Register of Systematic Reviews with the registration number CRD420251026145.

### 2.1. Search Strategy

To identify relevant studies, a systematic search was conducted in the DIALNET, ERIC, SCOPUS, Web of Science, MEDLINE, and PsycINFO databases. The search was performed up to 30 December 2024, ensuring a comprehensive review of the available literature.

Controlled MeSH terms were used in PubMed, while free-text terms were applied in other databases. The search equations were adapted for each database. For example, in PubMed, the following query was used: (“First aid” [All Fields] OR “cardiopulmonary resuscitation” [All Fields] OR “External Defibrillator” [All Fields]) AND (“physical education” [All Fields] OR “Health Education” [All Fields] OR “School Health Services” [All Fields]) AND “Methods” [All Fields].

Search terms were applied to “All Fields” in PubMed and “Title, Abstract, Keywords” in SCOPUS and Web of Science, adjusting for other databases as needed. The adapted search strategies for each database are provided in Supplementary Table S3. Additionally, the reference lists of included articles were manually reviewed to identify additional studies.

### 2.2. Study Selection and Data Extraction

Studies were selected according to the PICOS (participants, interventions, comparisons, outcomes, and study design) framework and met all of the following criteria: (i) studies conducted with secondary school students aged 11 to 18 years (compulsory secondary education); (ii) interventions focused on first aid teaching methodologies implemented within the PE subject; (iii) comparisons including traditional teaching methods, alternative active approaches (e.g., gamification, simulations, and cooperative learning), or no intervention groups; (iv) outcomes assessed included first aid-related knowledge acquisition (e.g., theoretical understanding of CPR steps and recognition of emergency situations), skill development (e.g., correct hand placement and compression depth and rate), knowledge retention (e.g., delayed post-tests), and/or public health competencies (e.g., confidence and willingness to act), which were measured using structured tools such as multiple-choice questionnaires, practical performance checklists, observational grids, and standardized perception scales; and (v) original peer-reviewed articles with randomized, quasi-experimental, or pre-experimental designs (e.g., pre-post studies without control groups), provided they implemented a structured intervention and assessed learning outcomes; articles must be published in English or Spanish.

The following studies were excluded from the review: (i) systematic reviews, meta-analyses, or narrative reviews; (ii) conference proceedings, abstracts, and studies without full-text availability; (iii) research conducted in non-educational or non-school settings; (iv) studies involving students with special educational needs; (v) studies that lacked sufficient methodological information for quality assessment. This included studies that did not report essential elements of study design, such as sampling methods, group allocation (randomized or not), the presence of a control group, blinding of assessors, or the validity and reliability of outcome measures. In practice, studies were excluded when the methodological description was too limited to be appraised using standardized tools such as the PEDro scale [30]; and (vi) gray literature, including theses, technical reports, and unpublished documents.

The study selection and the data management process were conducted in several phases. First, duplicate records were removed using Mendeley (version 1.19.8). Subsequently, the remaining records were imported into the Rayyan QCRI software (https://rayyan.ai), a free web-based tool designed to support systematic reviews through collaborative screening and data management [31]. Two authors (JMPE and MRM) independently screened titles and abstracts using the platform’s blinding function to reduce bias. Disagreements were resolved through discussion with a third author (SOG).

The data extraction process, conducted by the same two authors (JMPE and MRM), included information on author, year, sample size, age, study design, methodological description, duration of intervention, variables measured, and main results. This process was independently conducted by the two authors involved in the study selection.

### 2.3. Quality Assessment and Risk of Bias

The methodological quality of the included studies was assessed using the PEDro scale [30,32], a tool validated for quasi-experimental studies and controlled clinical trials. This instrument consists of 11 criteria, although only 10 are included in the final score. The first item assesses external validity (eligibility criteria), while items 2 to 9 relate to internal validity aspects such as randomization, allocation concealment, baseline comparability, blinding of participants, therapists, and assessors, as well as follow-up and intention-to-treat analyses. Items 10 and 11 evaluate statistical reporting and the presence of between-group comparisons. Each criterion was scored dichotomously (1 = criterion met; 0 = not met), resulting in a total score ranging from 0 (lowest quality) to 10 (highest quality), excluding item 1 from the total.

Studies were classified as follows: scores of 0–3 were categorized as poor, 4–5 as fair, 6–8 as good, and 9–10 as excellent, based on recent cut-off recommendations for the PEDro scale [30]. The assessment was conducted independently by two authors (JMPE and MRM), with discrepancies resolved by consensus or with the intervention of a third author (SOG).

## 3. Results

### 3.1. Data Search

The process followed for the selection of studies included in this systematic review is presented in Figure 1. The initial search identified 3107 studies across six databases. After removing 1346 duplicates using Mendeley, a total of 1761 records were screened by title and abstract, with 1633 studies excluded due to not meeting the eligibility criteria. A total of 128 full-text articles were assessed, of which 116 were excluded, mainly because they did not address first aid education in secondary schools. Ultimately, 11 studies were included in the systematic review.

### 3.2. Quality Assessment and Risk of Bias

According to the PEDro scale, six studies were classified as poor quality [3,5,18,33,34,35], and five studies were classified as fair quality [4,12,36,37,38]. No studies were rated as good or excellent quality.

The main sources of bias identified included the lack of randomization in eight studies [3,5,18,33,34,35,36,38] and the lack of allocation concealment in all eleven studies [3,4,5,12,18,33,34,35,36,37,38]; insufficient blinding was identified in seven studies [3,4,12,18,35,37,38], the absence of intention-to-treat analysis in all eleven studies [3,4,5,12,18,33,34,35,36,37,38], and inadequate statistical comparisons between groups in five studies [3,18,33,34,35]. No study was excluded due to a low PEDro score, but these factors were considered in the interpretation of the results.

The detailed quality assessment results are presented in a Table 1, displaying the scores obtained by each study across the different PEDro criteria.

These methodological limitations may affect the internal validity and reliability of the reported outcomes. The absence of randomization, identified in eight studies, including Lester et al. [33], Iserbyt [34], Martínez-Isasi et al. [3], Ming-Fen Tsai et al. [35], Miró et al. [18], Mpotos et al. [36], Van Raemdonck et al. [5], and Vetter et al. [38], introduces a potential selection bias, as pre-existing group differences unrelated to the intervention may influence the results.

Similarly, seven studies lacked blinding of participants and/or assessors, including Martínez-Isasi et al. [3], Ming-Fen Tsai et al. [35], Miró et al. [18], Otero-Agra et al. [4], Van Raemdonck et al. [37], Vetter et al. [38], and Watanabe et al. [12]. This methodological flaw may lead to an overestimation of the intervention’s effectiveness, particularly in studies relying on self-reported outcomes or subjective assessments.

Quasi-experimental designs without randomization or control groups are especially vulnerable to these risks and often report inflated effect sizes, especially in studies focused on short-term outcomes. Therefore, while most studies reported positive effects, these findings must be interpreted with caution, and future research should adopt more rigorous designs to validate the current evidence base.

Additionally, although findings were generally positive, they should be interpreted with caution given the limited number of included studies and the methodological heterogeneity observed.

### 3.3. Characteristics of the Studies and Teaching Methodologies Employed

A total of 11 studies were included [3,4,5,12,18,33,34,35,36,37,38], with a combined sample of 3069 secondary school students. In the study by Martínez-Isasi et al. [3], only data from students enrolled in the first year of compulsory secondary education were included (*n* = 206), in accordance with the predefined inclusion criteria. Participants from primary education were excluded from the analysis. The studies were conducted in Spain (*n* = 1306) [3,4,18], the United States (*n* = 535) [12,38], the United Kingdom (*n* = 41) [33], Belgium (*n* = 1212) [5,34,36,37], and Taiwan (*n* = 336) [35], reflecting a diversity of educational contexts and methodological approaches. Detailed information on each study’s context, methodology, and outcomes is presented in Table 2.

The publication years ranged from 1996 to 2022, allowing for an analysis of the evolution of first aid teaching methodologies in school settings. Early studies [33] focused on conventional CPR instruction, whereas more recent research [3] incorporated innovative approaches such as gamification and interactive learning.

The studies displayed considerable methodological heterogeneity, including quasi-experimental designs (*n* = 6; [3,4,18,35,36,37]), experimental designs (*n* = 3; [12,34,38]), and pre–post studies without control groups (*n* = 2; [5,33]). Sample sizes ranged from 41 to 593 participants, with most studies reporting a balanced gender distribution. However, two studies [34,36] reported gender differences in the willingness to perform CPR, with greater reluctance observed among female participants.

These gender-based discrepancies in CPR willingness have important implications for instructional design. As reported in the studies by Mpotos et al. (2017) [36] and Iserbyt (2016) [34], female students often expressed lower confidence and greater discomfort when performing mouth-to-mouth ventilation, particularly with strangers. Additionally, differences in physical strength and body weight may contribute to performance variability in chest compression depth and compression continuity.

To address these disparities, instructional programs should include strategies aimed at enhancing self-efficacy among female students, such as peer modeling, supportive feedback, and simulation scenarios with progressive levels of difficulty, while ensuring that all learners have access to practice opportunities adapted to their physical capabilities. Furthermore, incorporating guided discussions on psychological barriers and the importance of bystander intervention may help reduce hesitation and promote more equitable engagement in life-saving procedures.

The diversity in study designs and intervention durations posed a challenge when comparing the effectiveness of teaching methodologies. Quasi-experimental and experimental studies, such as those by Otero-Agra et al. [4], Miró et al. [18], Van Raemdonck et al. [5,37], Iserbyt [34], Ming-Fen et al. [35], Watanabe et al. [12], and Vetter et al. [38], provided more robust evidence, particularly when control groups or delayed post-tests were included.

In contrast, pre–post designs without control groups, as in Martínez-Isasi et al. [3] and Mpotos et al. [36], were more vulnerable to internal validity threats and less capable of capturing long-term outcomes. Although Lester et al. [33] followed an experimental approach, they focused solely on theoretical outcomes and did not include any follow-up assessments.

Outcome measures also varied considerably, ranging from written knowledge tests to practical CPR assessments and self-reported confidence levels. While Table 2 details these methodological differences, the heterogeneity across studies limits comparability and prevents meaningful quantitative synthesis. Therefore, generalized conclusions should be drawn with caution.

To complement the methodological analysis, Table 3 summarizes the follow-up assessments and long-term retention outcomes reported in the included studies. It highlights the timeframes and types of outcomes evaluated, such as theoretical knowledge, CPR skills, or willingness to act, and offers insights into the persistence of learning over time. This information adds context to the discussion on the long-term effectiveness of first aid teaching methodologies.

The duration and frequency of interventions varied notably. Program lengths ranged from a single 45–60 min session [33,34,36] to extended interventions lasting up to seven weeks [18]. While some studies delivered one or two sessions per week [3,34,37], others implemented more intensive weekly schedules [12,38]. Overall, longer programs were consistently associated with greater long-term skill retention and improved performance in emergency scenarios [4,5,35].

The evaluated methodologies were grouped into three main categories: (a) traditional methods, such as expository theoretical classes and instructor-led demonstrations [18,33]; (b) active methods, including simulations, role-playing, gamification, and peer evaluation [3,4,5,34,36,38]; and (c) technology-based methods, such as virtual reality, mobile applications, and online platforms [12,35].

The effectiveness of these methodologies varied depending on the instructional approach and the assessment tools used. Active methods improved knowledge acquisition, which was primarily assessed using multiple-choice questionnaires, by 10.2% [3] to 24.7% [36], and practical skill development, which was measured via manikin-based checklists and observational grids, by 21.5% [34] to 39.8% [4] compared to traditional approaches. Technology-based strategies enhanced knowledge acquisition by 5.8% [12] to 12.6% [3] and skill acquisition by 7.3% [35] to 14.2% [38].

Regarding assessment strategies, theoretical knowledge was typically evaluated using multiple-choice tests [33,34,35,36], while practical competencies were assessed through CPR performance checklists, manikin-based simulations, or real-time observation tools [3,5,34,38]. Several studies employed both types of instruments to provide a comprehensive assessment of learning outcomes [4,18].

Despite the variety of instruments used across studies, few provided detailed information on the psychometric properties of the tools employed. Some studies used validated instruments, such as standardized multiple-choice tests to assess theoretical knowledge (Watanabe et al., 2017 [12], and Ming-Fen et al., 2019 [35]) or QCPR-equipped manikins that provided feedback on compression quality (Otero-Agra et al., 2019 [4]; Iserbyt, 2016 [34]; Vetter et al., 2016 [38]), which offer objective and replicable data.

However, many studies relied on ad hoc or non-validated instruments, particularly for measuring confidence, willingness to act, or overall skill performance, without reporting internal consistency, test–retest reliability, or validity indices. This heterogeneity limits the comparability of findings and introduces potential measurement bias.

Moreover, our review identified that active methodologies did not consistently outperform traditional ones, and part of this variability may stem from inconsistencies in outcome assessments. We therefore recommend that future studies employ standardized and psychometrically validated tools, such as validated CPR knowledge questionnaires, QCPR systems, or structured observer-based checklists with interrater reliability reporting, to improve methodological transparency and enable more accurate cross-study comparisons.

Notably [4], implemented real-time visual feedback led to a significant improvement in compression quality, while [35] integrated mobile applications allowed researchers to monitor CPR performance with greater precision.

**Figure 2:**
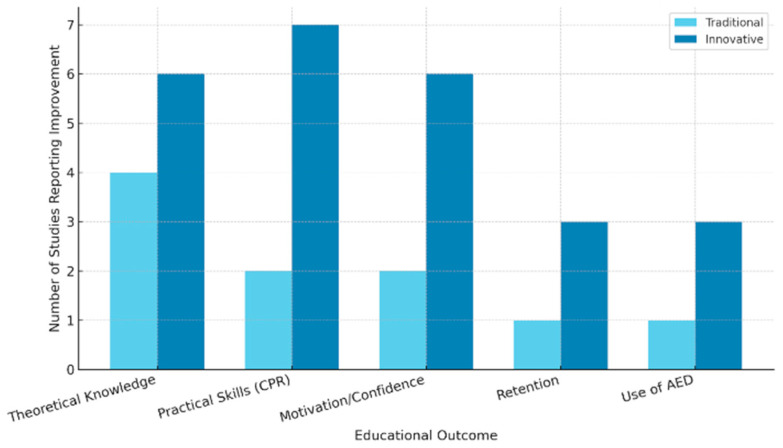
Summary of the main educational outcomes reported in the included studies, categorized by teaching methodology (traditional vs. innovative). The *Y*-axis indicates the number of studies reporting improvement in each outcome. Innovative methodologies include peer instruction, gamification, audiovisual feedback, and flipped learning approaches.

Figure 2 presents a visual summary of the number of studies reporting improvements in each educational outcome, categorized by type of teaching methodology. This figure allows for a clearer comparison between traditional and innovative approaches, highlighting the greater consistency of positive outcomes in studies that employed active or technology-enhanced strategies.

Although all studies assessed outcomes related to knowledge acquisition, practical skills, or retention, the specific constructs and measurement tools varied considerably. Some studies evaluated theoretical understanding using multiple-choice tests, while others used open-ended questions or custom-developed instruments. Practical performance was measured using manikin-based checklists or observational grids, applying varied criteria and scoring thresholds. Due to this heterogeneity, direct comparisons should be interpreted with caution and were addressed descriptively rather than statistically.

## 4. Discussion

This systematic review aimed to examine first aid teaching methodologies in secondary school PE and assess their impact on students’ emergency response skills.

A total of 11 studies were included, involving 3069 students aged 11 to 18 years; one study [12] (*n* = 41) did not report gender distribution [12]. The analyzed interventions focused primarily on CPR training, along with other first aid techniques such as AED use, airway management, and bleeding control [36].

The results confirm that active methodologies, including gamification, cooperative learning, and technology-based approaches, significantly enhance first aid instruction in this educational context [4,12,39]. These strategies not only improve knowledge acquisition and the development of practical skills but also boost students’ confidence in performing first aid and strengthen their ability to respond effectively to emergencies [17]. The integration of these pedagogical approaches holds important implications for both school safety and public health, reinforcing the case for their inclusion in the educational curriculum [4,8,20,40,41,42].

The analysis of the included studies demonstrates that teaching strategies based on hands-on practice and interactive learning enhance long-term knowledge retention. Studies such as those by Otero-Agra et al. [4] and Bray et al. [17] support this finding, highlighting the effectiveness of interactive and practical learning environments. The authors of [4] found that students trained through cooperative learning exhibited 30% greater confidence in executing first aid procedures compared to those taught using traditional methods [1]. Moreover, gamification has proven to be an effective strategy for improving student motivation and engagement, fostering greater involvement in safety and emergency preparedness education [4,39,43,44].

The relevance of these strategies has been highlighted in previous reviews, such as Martínez-Isasi et al. [3], emphasizing how the combination of interactive practice and real-time feedback can optimize the teaching–learning process [3,4,6,39].

The impact of these methodologies extends beyond knowledge retention to the applicability of acquired skills. Simulation-based instruction and peer evaluations enhance CPR quality, ensuring the correct execution of maneuvers and reducing variability in student performance [45,46,47].

Additionally, technology-based approaches, such as interactive applications and real-time feedback, have been shown to be valuable tools for optimizing learning, enabling performance monitoring and individualized instructional adjustments [48,49].

However, the effectiveness of these methodologies largely depends on the quality of their implementation and the level of teacher training [25,50,51,52,53], highlighting the need for specialized professional development to ensure their proper application in the classroom.

In comparison with previous research [1,8,11,54,55,56], this review further supports the effectiveness of active methodologies in first aid education [1]. Studies such as the review by Martínez-Isasi et al. [3] have documented significant improvements in knowledge retention and the practical application of CPR techniques when experience-based learning strategies are employed. However, some studies suggest that traditional teaching methods remain effective in certain contexts, particularly in resource-limited environments or settings with restricted access to educational technology [25,37,52]. These differences highlight the importance of adapting teaching methodologies based on the educational context and student characteristics. [17,36,49,52].

While active approaches generally yield positive results, their effectiveness and feasibility vary depending on the specific method employed. For instance, gamification has been shown to enhance student engagement and compression quality in some studies (e.g., Otero-Agra et al. [4]), whereas peer instruction and audiovisual feedback may better support skill retention and self-assessments (e.g., Mpotos et al. [36], Iserbyt [34], and Van Raemdonck et al. [5]).

Recent guidelines also emphasize the value of combining multiple strategies, such as spaced learning, real-time feedback devices, and simulation-based practice, to optimize outcomes in CPR education [10]. Moreover, the instructor’s training plays a pivotal role in the success of these methodologies, as evidenced by school-based programs in which PE teachers received prior pedagogical and technical preparation [9].

However, not all active methods consistently outperform traditional ones across all domains or populations. Therefore, future research should directly compare different active strategies to identify those best suited to specific educational contexts, student profiles, and instructional objectives. Recognizing this methodological diversity is essential to avoid overgeneralization and to promote context-sensitive pedagogical design.

Although this systematic review provides relevant findings, several methodological limitations must be considered. The heterogeneity in study designs, variability in evaluation instruments, and differences in intervention duration make direct comparisons of the results challenging. Additionally, most studies only assessed short-term outcomes, preventing an evaluation of long-term knowledge retention.

In this regard, future research should incorporate longitudinal designs to analyze the long-term effectiveness of these methodologies and their applicability in diverse educational settings [57]; researchers have emphasized the importance of periodic follow-ups to determine the sustained impact of active methodologies over time. To address this issue, comparative studies between active and traditional methodologies with follow-up assessments at six months and one year post-intervention are recommended.

An additional relevant limitation is the short-term follow-up applied in most of the included studies. The majority of interventions were assessed only immediately after their implementation, with limited evidence on the medium- or long-term retention of knowledge and skills. This restricts our understanding of the durability and real-world impact of first aid training programs. Previous research has highlighted the importance of delayed post-tests and long-term evaluations to assess the persistence of learning outcomes over time. For example, Watanabe et al. [12] demonstrated that re-education sessions conducted two to four months after initial training significantly improved knowledge retention compared to one-time interventions.

Similarly, the European Resuscitation Council recommends annual retraining as a feasible and effective strategy to maintain basic life support (BLS) skills among school-aged students [10]. Therefore, future studies should adopt longitudinal designs with follow-up assessments at 3, 6, and 12 months to evaluate both knowledge and skill retention, as well as the transfer of learning to real-life emergency situations. The incorporation of delayed post-tests and the use of validated assessment tools will be essential to produce robust, reliable, and generalizable evidence.

To further support this discussion, Table 3 presents a summary of the follow-up assessments conducted in the included studies. Of the 11 studies, 9 incorporated follow-up evaluations ([5,12,33,34,35,37,38]), with timeframes ranging from 9 days to 12.5 months after the intervention. Most assessments focused on practical CPR skills, while fewer studies evaluated theoretical knowledge, anxiety, or willingness to act.

The findings were mixed: while some studies reported a decline in performance in the absence of continued practice, others, particularly those incorporating gamification, repeated feedback, or creative engagement strategies, demonstrated greater retention over time.

Another key limitation is the lack of standardized evaluation criteria. The review by Van Raemdonck et al. [5] highlighted that the absence of homogeneous measurement tools makes it difficult to compare results across studies. To enhance the validity and comparability of future research, it is essential to establish standardized protocols for assessing first aid learning outcomes. The development of validated measurement instruments would contribute to greater study replicability and enable the conduct of more robust meta-analyses.

The findings of this review have important implications for both educational practice and public health. The integration of active methodologies in first aid instruction in PE can significantly improve student preparation to respond to emergencies, reducing reaction time and increasing survival rates in critical situations [58]. Additionally, teacher training in these instructional strategies is crucial for ensuring effective classroom implementation and maximizing the impact on student learning. Previous studies suggest that continuous teacher training in first aid and active methodologies enhances teaching quality and strengthens instructor confidence in delivering these competencies [16].

Enhancing teacher training is essential for the effective implementation of active and student-centered instructional strategies in first aid education. Empirical evidence shows that instructors who receive pedagogical training focused on simulation, gamification, and feedback techniques demonstrate greater confidence, methodological consistency, and improved student engagement [4,9].

Moreover, the integration of structured rehearsal, access to validated instructional materials, and peer collaboration further strengthens the fidelity of innovative methodologies in CPR instruction [10]. International guidelines emphasize that the quality of CPR training strongly depends on the educators’ competence and the application of educational strategies grounded in learning theories, formative feedback, and spaced repetition [10].

Therefore, embedding continuous professional development (CPD) programs for teachers within school systems is a key factor in enhancing both the instructional quality and the learning outcomes of CPR education at the school level [34,36].

In resource-limited educational settings, active methodologies can be effectively adapted using minimal resources. Peer instruction led by PE teachers has demonstrated positive outcomes in both knowledge acquisition and CPR skills, even among younger students when age-appropriate methods are applied [3]. Additionally, gamified strategies, particularly those incorporating visual feedback, enhance student motivation and improve compression quality [4]. These low-cost approaches, including team-based activities, role-playing, and printed instructional guides, provide feasible and scalable solutions for schools with limited access to technology or external personnel.

To facilitate the integration of first aid instruction into PE curricula, we propose a set of practical implementation guidelines synthesized from the findings of the included studies and aligned with international recommendations [4,5,9].

Although intervention formats varied considerably, the most effective programs generally lasted between 90 and 180 min and were delivered across multiple sessions, typically spanning 2 to 6 weeks.

Recommendations regarding equipment requirements, session frequency, and low-cost adaptations are summarized in Table 4. These guidelines are intended to support teachers and school planners in designing feasible and scalable interventions across diverse educational settings.

First aid education in schools should be considered a key public health strategy, as its implementation at the secondary level can reduce morbidity and mortality associated with medical emergencies [3,6,25]. Integrating these programs into educational and health policies would enable broader population coverage and more standardized preparedness for emergency response. Previous research has demonstrated that early first aid training not only improves individual emergency response capacity but also creates a multiplier effect within the community, as trained students can transfer knowledge to their families and social circles [59]. In this regard, strengthening first aid instruction in PE represents a public health intervention with both short- and long-term impacts, justifying its inclusion in curricular plans as an essential component of safety and prevention education [3].

## 5. Conclusions

This systematic review confirms that active methodologies, such as gamification, cooperative learning, and simulations, are effective in teaching first aid in secondary school PE. These strategies enhance knowledge retention, increase confidence in performing first aid procedures, and strengthen emergency response capabilities.

From a public health perspective, first aid education in secondary schools represents a key intervention to reduce morbidity and mortality in school and sports environments. Its inclusion in the educational curriculum enhances safety and promotes a culture of rapid emergency response.

Despite its benefits, methodological challenges remain, including study heterogeneity and the lack of standardized protocols. Future research should focus on analyzing the long-term effectiveness of these methodologies and developing guidelines to facilitate their implementation in PE. The consolidation of these approaches in educational and health policies would contribute to better population preparedness for emergencies and foster safer school environments.

## Figures and Tables

**Table 1:** Quality assessment with the PEDro scale of the included studies.

First Author, Publication Year	Criterion											Total Quality Scores
	1	2	3	4	5	6	7	8	9	10	11	
Iserbyt (2016) [34]	1	0	0	0	0	0	1	1	0	0	1	3
Lester et al. (1996) [33]	1	0	0	0	0	0	1	1	0	0	1	3
Martínez-Isasi et al. (2022) [3]	1	0	0	1	0	0	0	1	0	0	1	3
Ming-Fen et al. (2019) [35]	1	0	0	0	0	0	0	1	0	0	1	2
Miró et al. (2005) [18]	1	0	0	0	0	0	0	1	0	0	1	2
Mpotos et al. (2017) [36]	1	0	0	0	0	0	1	1	0	1	1	4
Otero-Agra et al. (2019) [4]	1	1	0	1	0	0	0	1	0	1	1	5
Van Raemdonck et al. (2014) [37]	1	1	0	1	0	0	0	0	0	1	1	4
Van Raemdonck et al. (2017) [5]	1	0	0	0	0	0	1	0	0	1	1	3
Vetter et al. (2016) [38]	1	0	0	1	0	0	0	1	0	1	1	4
Watanabe et al. (2017) [12]	1	1	0	1	0	0	0	1	0	1	1	5

1. Specification of eligibility criteria; 2. random allocation; 3. allocation concealment; 4. comparable baseline: groups were similar at the start regarding the most important prognostic indicators; 5. patient “blinded”; 6. therapist “blinded”; 7. assessor “blinded”; 8. subject follow-up (at least 85% follow-up); 9. intention-to-treat analysis; 10. statistical comparisons between groups; 11. measure of variability and point estimates. Criterion 1 is not considered in the total sum.

**Table 2:** Characteristics of the 11 included studies.

Author, Year	Sample Characteristics (*n*, Gender, Age)	Study Design	Intervention Methodology	Duration of Intervention	Outcomes Measured	Main Results
Lester et al. [33] (1996)	EG: *n* = 17 (7 ♀); 13.9 ± 0.6 y (mean age not specified) CG: *n* = 24 (17 ♀); 13.8 ± 0.5 y (mean age not specified)	Pre- experimental (pre-post without control group)	No CG.EG: ERC-based CPR training delivered by PE teachers using BHF video, segmented instruction, peer practice with manikins, and ethical discussion. EG1: Same CPR training protocol delivered by peer instructors previously trained by the same teachers.	6 weeks 1 session/week, 50 min/session (300 min total)	Theoretical knowledge (multiple-choice test), CPR skills (checklist evaluation), and CPR confidence (perception questionnaire)	Effect: Yes (knowledge); no (CPR skills and confidence). Result: A total of 58.5% scored ≥70% in knowledge; critical CPR steps poorly executed; low real-life confidence (no *p*-values reported).
Iserbyt [34] (2016)	SG. *n* = 313 (133 ♀); 12–18 y (mean age not specified)	Pre- experimental (pre–post without a control group)	No CG; EG: CPR training delivered by PE teachers using a reciprocal peer-learning model, instructional videos on iPads, and peer practice alternating trainer/trainee roles with manikins.	1 single session 50 min/session	Willingness to perform BLS (questionnaire), fear of performing BLS (questionnaire), and BLS quality (evaluation using a Laerdal manikin)	Effect: Yes (willingness in boys, fear in girls, and BLS–willingness correlation). Result: Boys improved willingness to act on family (49% to 58%, *p* = 0.01); girls showed reduced fear of ventilations (*p* < 0.01); higher initial willingness correlated with better BLS performance (*p* = 0.02).
Martínez-Isasi et al. [3] (2022)	EG: *n* = 206 (93 ♀); 11.3 ± 0.5 y (mean age not specified) ^1^ CG: No control group	Pre- experimental (pre–post without a control group)	No CG; EG: Two 50 min BLS sessions delivered by PE teachers during regular PE classes to first year ESO students. Session 1: Theoretical content using age-appropriate materials. Session 2: Practical CPR training using adult-size manikins with visual/audio feedback.	2 sessions, 50 min each (100 min total)	Theoretical knowledge (test), execution of the BLS sequence (simulation), and CPR quality (initiation time, compression fraction, depth, rate, chest recoil)	Effect: Yes (knowledge, continuity, depth, and rate); no (recoil and full BLS sequence). Result: Knowledge improved from 23.3% to 77.0% (*p* < 0.001); 28.5% completed full BLS sequence (*p* = 0.030); improvement in continuity, depth, and rate (*p* < 0.001); recoil decreased (*p* < 0.001).
Ming-Fen et al. [35] (2019)	EG: *n* = 336 (189 ♀); (12–13 y) CG: No control group	Pre- experimental (pre–post without a control group)	No CG; EG: A 50 min CPR/AED education session conducted by certified emergency trainers, including theoretical instruction (lecture and video demonstration) and practical training with manikins. Students practiced hands-on CPR skills individually.	1 single session, 50 min	Theoretical knowledge (test), correct emergency response actions (simulation), and willingness to perform CPR (questionnaire)	Effect: Yes (knowledge, actions at home/public, and willingness); no (confidence and anxiety). Result: Significant improvement in emergency knowledge (*p* < 0.001) and correct actions at home (*p* < 0.01) and in public (*p* < 0.001); willingness to act increased from 9.5% to 42.6% (*p* < 0.001); 51.5% reported greater confidence and 44.9% reduced anxiety (no *p*-values reported).
Miró et al. [18] (2005)	SG: *n* = 250 (120 ♀); 14.9 ± 0.9 y (mean age not specified) (SG: Single group)	Pre- experimental (pre-post without control group)	No CG; EG: Sessions 1–4 (theory, 4 h): Audiovisual materials; Sessions 5–6 (practice, 4 h): Training included manikin-based CPR practice and simulation-based scenarios; Session 7 (1 h): Assessment: 20-question pre–post-test.	7 weeks 1 session/week 60 min/session (420 min total)	Theoretical knowledge (multiple-choice test, 20 questions), CPR skills (mannequin practice evaluated by an observer), opinion, and satisfaction (post-training survey)	Effect: Yes (knowledge and CPR skills). Result: Scores improved from 8.5 to 13.5 points out of 20 (*p* < 0.001); greater gain among students without prior training.
Mpotos et al. [36] (2017)	EG: *n* = 265 (111 ♀); 12–18 y (mean age not specified) CG: No control group	Pre- experimental (pre–post without a control group)	No CG; EG: BLS training session (45 min) delivered by PE teachers using reciprocal peer-learning with iPads. Students practiced CPR (30:2 compressions/ventilations) with manikins, alternating trainer and trainee roles in pairs, and conducted peer assessments.	1 single session, 45 min (20 min practice, 10 min evaluation)	Chest compression quality (depth, rate, and recoil) in relation to age and physical characteristics	Effect: No (training); yes (age/sex-related performance differences). Result: Only 18% of the youngest reached the correct depth; compression quality improved with age and strength (*p* < 0.05); males had better recoil and technique.
Otero-Agra et al. [4] (2019)	EG1 (GAM): *n* = 151 (♀ no especificado); 13.6 ± 1.0 y EG2 (EVA): *n* = 140 (♀ no especificado); 13.4 ± 1.2 y EG (VFC): *n* = 109 (♀ no especificado); 13.4 ± 1.1 y CG (TC): *n* = 89 (♀ no especificado); 13.6 ± 1.2 y	Quasi-experimental (randomized-block design)	EG1 (GAM): Gamification-based CPR training (playing in teams), visual, and instructor feedback; compulsory curricular activity, non-tested. EG2 (EVA): CPR training with visual and instructor feedback; compulsory curricular activity with subsequent individual evaluation (test). EG3 (VFC): Non-compulsory CPR training with visual and instructor feedback; non-tested academic activity. CG (TC): Traditional non-compulsory CPR training, only instructor feedback; non-tested academic activity.	1 single session, 50 min (15 min theory and 35 min practice)	CPR quality (QCPR %), total number of compressions, compression depth and rate, correct release rate, and compression time	Effect: Yes (CPR quality and compression depth). Result: The GAM group reached 89.56% CPR quality (*p* < 0.001); 93.4% had compressions >50 mm (*p* < 0.001); no differences vs. EVA.
Van Raemdonck et al. [37] (2014)	EG1: *n* = 43 (0 ♀); 15–16 y EG2: *n* = 44 (0 ♀); 15–16 y EG3: *n* = 36 (0 ♀); 15–16 y EG4: *n* = 42 (0 ♀); 15–16 y	Quasi-experimental (group randomized trial)	EG1: CPR manikin + teacher instruction EG2: CPR manikin + video instruction EG3: Foam + plastic bag + peer practice + teacher instruction EG4: Foam + plastic bag + peer practice + video instruction	1 single session 50 min/session (6 min hands-on time)	CPR skills (practical test using SkillReporting Software, version 2.2.1) and knowledge retention at 6 months	Effect: No. Result: Low CPR quality across groups; only 18% achieved correct compression depth, and 32% achieved correct ventilation volume; skills declined after 6 months (no *p*-values reported).
Van Raemdonck et al. [5] (2017)	EG: *n* = 41 (29 ♂, 12 ♀); 15–17 y CG: No control group	Pre- experimental (pre–post without a control group)	No CG; EG: Intervention with self-directed e-learning CPR training (flipped classroom). Participants trained autonomously for 6 weeks using an online platform (theoretical content, instructional videos, and micro-simulation scenarios), without physical manikin practice.	6 weeks, self-learning with no fixed sessions, total practice time: 39–71 min depending on performance	Theoretical knowledge (test), CPR and AED skills (manikin-based evaluation), compression quality (depth, rate), and AED use (electrode placement and safety)	Effect: Yes (knowledge and AED use); no (airway and compression depth). Result: A total of 18% reached correct depth; certified students showed better compressions (*p* = 0.002); all required hands-on practice to master skills.
Vetter et al. [38] (2016)	EG: *n* = 230 (63% ♀); 16.1 ± 1.4 y CG: *n* = 182 (64.3% ♀); 15.8 ± 1.2 y	Quasi-experimental (prospective controlled trial without randomization)	CG: Standard CPR/AED training delivered by health teachers with theoretical and practical instruction. EG: Same CPR/AED training plus student-developed educational programs (innovative peer-teaching strategies, multimedia methods, and competitive CPR/AED Olympics event)	1 single session ~60 min/session (not specified)	Theoretical knowledge (test), CPR and AED skills (manikin-based evaluation), and willingness to act (scenario-based questionnaire).	Effect: Yes (knowledge, CPR/AED skills, and retention). Result: Significant improvement in knowledge and skills in both groups (*p* < 0.001); the experimental group retained skills better (88% vs. 79%, *p* < 0.001); 93.1% success in the simulation test.
Watanabe et al. [12] (2017)	EG1: *n* = 18 (sex not reported); (13–14 y) EG2: *n* = 23 (sex not reported); (13–14 y)	Randomized controlled trial (RCT)	EG1: Single 45 min BLS/AED session provided by AHA-certified instructors (theoretical instruction, demonstration, hands-on practice with manikins, and AED). EG2: Identical initial training plus an additional re-education session after 2 months.	1 single session, 45 min; re-education in the experimental group at 2 months, 45 min	Theoretical knowledge (test), CPR and AED skills (manikin-based practical evaluation), and retention at 2 and 4 months	Effect: Yes (knowledge and skills); partial (retention). Result: Knowledge and skills improved after the initial session (*p* < 0.001); re-education improved knowledge retention at 4 months (*p* = 0.0097) but not skills; AED use more accurate in the re-educated group.

AED: automated external defibrillator; BLS: basic life support; CG: control group; CPR: cardiopulmonary resuscitation; EG: experimental control; ESO: compulsory secondary education; EVA: training with evaluation; GAM: gamification with visual feedback and competence; QCPR: CPR quality (%); RCT: randomized controlled trial; SG: signal group; TC: traditional training without visual feedback; VFC: training with visual feedback without evaluation; y: years. ^1^ Only students in their first year of compulsory secondary education were included.

**Table 3:** Summary of follow-up assessments and long-term retention outcomes in the included studies.

Author, Year	Follow-Up Assessment	Timeframe	Type of Outcome Measured	Retention Findings
Lester et al. [33] (1996)	Yes	9 days	Theoretical knowledge and practical CPR skills	Skills declined rapidly despite good theoretical knowledge. Errors included failure to call emergency services or incorrect compressions.
Iserbyt [34] (2016)	Yes	1 week	Practical CPR performance and real-life willingness to act	Slight improvement in CPR performance. Limited effect on overall willingness to act, except among boys with family members.
Martínez-Isasi et al. [3] (2022)	No	N/A	N/A	N/A
Ming-Fen et al. [35] (2019)	Yes	1 week	Theoretical knowledge, willingness to act, and perceived anxiety	Increased confidence and reduced anxiety, though 57.4% still hesitated to apply CPR.
Miró et al. [18] (2005)	No	N/A	N/A	N/A
Mpotos et al. [36] (2017)	Yes	1 week	Practical skill: compression depth	Compression performance varied by age, sex, and weight. Boys aged 16–18 reached 90% correct depth.
Otero-Agra et al. [4] (2019)	Yes	1 week	CPR skills (quality of compressions, depth, and rhythm)	Gamification led to better compression quality and depth than traditional methods.
Van Raemdonck et al. [37] (2014)	Yes	6 months	Practical CPR skills (depth, rhythm, and ventilation volume)	Retention maintained after 6 months. Over 50% achieved the correct compression rate.
Van Raemdonck et al. [5] (2017)	Yes	6 weeks	Compression skills and AED use	Only 18% reached adequate compression depth. Self-training aided theory but not practical skills.
Vetter et al. [38] (2016)	Yes	12.5 months	Psychomotor skills (depth, rhythm, and hands-off time) and theoretical knowledge	The experimental group retained CPR skills; the control group showed significant decline.
Watanabe et al. [12] (2017)	Yes	4 months	Theoretical knowledge and practical CPR and AED skills	Knowledge and skill retention observed. Retraining improved AED use and response time.

AED: automated external defibrillator; CPR: cardiopulmonary resuscitation; N/A: not applicable.

**Table 4:** Practical guidelines for implementing first aid education in Physical Education contexts.

Aspect	Recommendation
Session frequency	1–2 sessions per week over 2–6 weeks [4,5]
Total duration	90–180 min delivered across multiple sessions [12,34]
Minimum equipment	1 CPR manikin per 3–4 students [9,10,16]
Optional resources	AED trainer, QCPR manikins with feedback, and gamified materials [3,38]
Resource-limited tips	Peer instruction, printed checklists, role-play, and recycled props [4,10]

AED: automated external defibrillator; CPR: cardiopulmonary resuscitation; QCPR: CPR training manikin with real-time performance feedback (depth, rate, recoil, etc.).

## Data Availability

No new data were created or analyzed in this study. Data sharing is not applicable to this article.

## Supplementary Materials

The following supporting information can be downloaded at: https://www.mdpi.com/article/10.3390/healthcare13101112/s1, Table S1: PRISMA guidelines checklist for the present systematic review. Table S2: PRISMA 2020 for Abstracts Checklist. Table S3: Search terms and search strategies to each database. Ref. [60] is cited in the Supplementary Materials.
